# A Modified 2D Multiresolution Hybrid Algorithm for Ultrasound Strain Imaging

**DOI:** 10.1155/2017/2856716

**Published:** 2017-12-20

**Authors:** Jibing Wu, Yang Jiao, Zhile Han, Jie Xu, Yaoyao Cui

**Affiliations:** ^1^Suzhou Institute of Biomedical Engineering and Technology, Chinese Academy of Sciences, Suzhou, Jiangsu 215163, China; ^2^University of Chinese Academy of Sciences, Beijing 100049, China

## Abstract

Ultrasound elastography is an imaging modality to evaluate elastic properties of soft tissue. Recently, 1D quasi-static elastography method has been commercialized by some companies. However, its performance is still limited on high strain level. In order to improve the precision of estimation during high compression, some algorithms have been proposed to expand the 1D window to a 2D window for avoiding the side-slipping. But they are usually more computationally expensive. In this paper, we proposed a modified 2D multiresolution hybrid method for displacement estimation, which can offer an efficient strain imaging with stable and accurate results. A FEM phantom with a stiffer circular inclusion is simulated for testing the algorithm. The elastographic contrast-to-noise rate (CNRe) is calculated for quantitatively comparing the performance of the proposed algorithm with conventional 1D elastography using phase zero estimation and the 1D elastography using downsampled (d-s) baseband signals. Results show that the proposed method is robust and performs similarly as other algorithms in low strain but is superior when high level strain is applied. Particularly, the CNRe of our algorithm is 15 times higher than original method under 4% strain level. Furthermore, the execution time of our algorithm is five times faster than other algorithms.

## 1. Introduction

Mechanical properties of soft tissue have been used as an important indicator of several diseases, such as breast [1, 2], liver, and prostate cancer [3, 4]. Generally, the carcinoma tissue is stiffer than the surrounding normal tissues. Palpation, as the standard procedure during physical examination, is used to touch and feel tissue below the surface of the body, while its accuracy is limited by the depth of measurement [5]. Ultrasound elastography is a relative new imaging modality for the clinical evaluation of elastic properties of soft tissue using ultrasound. It was first referred to as elastography by Ophir et al. in 1991 [6]. It became a very hot research focus of medical ultrasound in the last two decades [7, 8]. Major medical imaging device companies have launched their ultrasound products with elastography or shear wave imaging modalities [7–10]. Clinical trials have been conducted for various clinical applications with promising results.

Ultrasound elastography has been developing into an effective method in cancer diagnosis due to its capability and simple implementation [11]. The basic steps of the technique are as follows: (1) the biological tissue is compressed by contact or noncontact way; (2) backscattered radio frequency (RF) signals before and after tissue compression are collected, respectively, by ultrasonic transducer; (3) the tissue displacement estimation algorithm is applied to estimate the displacement field from the RF signals; and (4) the strain field is reconstructed from the displacement field by strain estimation algorithm.

Accurate estimation of tissue displacement is a very important step in ultrasound elastography. Different methods were proposed in the last two decades. Majority of these methods use correlation technique in time domain or the phase domain for displacement estimation [6, 12–14]. In time domain, several matching operators like correlation coefficients, the sum of squared differences (SSD), and the sum of absolute differences (SAD) are employed to finding the optimal matching in the pre- and postcompression RF signals [15, 16]. Since the computational cost of the matching operators in time domain is large, phase-based methods like phase zero estimation, which have higher computational efficiency than time-domain methods, have been implemented in clinical ultrasound system for real-time strain imaging [13, 17]. Both 1D and 2D elastography methods can obtain displacements field, but the 1D estimation algorithms only consider axial displacements in tissue. They have difficulty in obtaining precise results in complex tissue environment, especially under high compression conditions. In general, the RF data sequence will be shifted laterally because of the lateral displacement in tissue. In order to reduce the errors due to lateral motion in 1D elastography, 2D elastography algorithms like hybrid displacement estimation method and a modified block matching method have been proposed [18, 19]. These methods consider both axial and lateral displacement; they expand the 1D window to a 2D window to avoid the side-slipping of the 1D window, which can greatly improve the precision of estimation result in high compression situation, but 2D elastography is usually more computational expensive than 1D elastography.

In this paper, we proposed a modified 2D multiresolution hybrid method for displacement estimation, which can offer an efficient strain imaging with stable and accurate results. To test the algorithm, a heterogeneous computational phantom is simulated using finite element model (FEM), with a rectangle background containing a stiffer circular inclusion. The synthetic RF data are generated from Filed II software [20]. We compare the result with three different algorithms and show a great improvement of our method in ultrasound elastography.

## 2. Methods

In order to make a tradeoff between speed and accuracy, we proposed a method using modified 2D multiresolution hybrid elastography. Preprocessing procedure is first applied to the raw RF data to obtain envelopes and baseline signals at different resolutions. Chen et al. proposed a hybrid displacement estimation method, which applied 3-level estimation based on cross-correlation and weighted phase separation (WPS) [18]. We suggest processing coarse estimation also on 3-level cross-correlation on sampled RF data by different sampling rate. And then a fine estimation is carried out on the whole frame RF date by phase zero-crossing method [15], which uses the results of the coarse estimation as the input to improve the resolution and accuracy.

### 2.1. Preprocessing

The analytic signals of predeformation and postdeformation radio frequency (RF) signals are obtained by applying Hilbert transformation to the raw RF data. The baseband signals can be calculated by demodulating the analytic signals with a carrier wave exp⁡(*jw*_0_*t*), where *w*_0_ denotes the modulation frequency. The modulation frequency *w*_0_ should be chosen close to the transducer's center frequency.

The baseband signals are downsampled at different downsample rates. The downsample rates should satisfy the Nyquist sampling condition. The downsampled baseband signals are then converted into amplitude and phase data using FFT. The amplitude data at different scales are used in corresponding coarse to fine estimation, but the phase data are only used for fine estimation.

### 2.2. Coarse Estimation

#### 2.2.1. Level 1 Search

**Figure 1:**
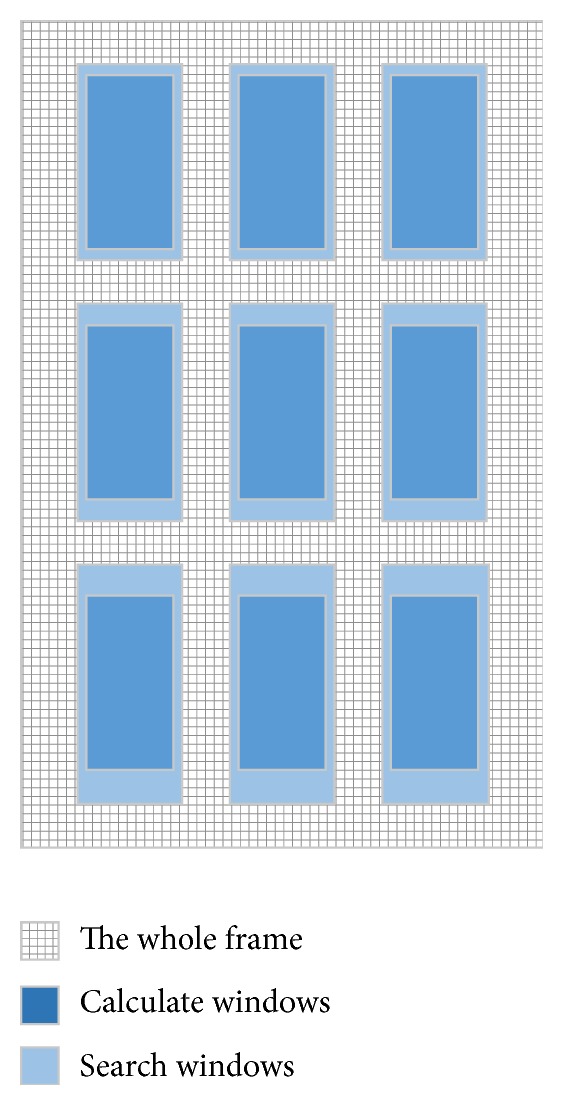
Over of the level 1 search, the calculate windows and search windows are equally distributed.

Nine evenly distributed windows have been selected in the coarsest scale of the predeformation frame (see Figure 1). Displacements are calculated in these 9 windows by finding the highest correlation coefficient between predeformation and postdeformation frames in a search window [19, 21]. The correlation coefficient can be expressed as

(1) Rdx,dy=∑x,y∈TAx,y−A¯Bx+dx,y+dy−B¯∑x,y∈TAx,y−A¯2∑x,y∈TBx+dx,y+dy−B¯2,

where *d*_*x*_ is the lateral displacement, *d*_*y*_ is the axial displacement, *A* and *B* denote the envelope data from predeformation and postdeformation frame, $\bar{A}$ and $\bar{B}$ are the averages of *A* and *B*, and *T* is the window size. Since computational cost at this level is relatively cheap, the size of search windows can be set large enough, and in order to reduce the errors in this level, we do not use multiresolution search method mentioned by Chen in this level. In addition, the size of the search windows in the lower rows is selected bigger than in the upper row since displacement will be accumulated with respect to depth. The output of level 1 search is nine axial-lateral displacement estimations within the nine search windows.

#### 2.2.2. Level 2 Search

Level 2 search is performed at a finer scale than level 1 search. Seven by 11 evenly distributed calculate windows are selected on the predeformation frame. The size of the calculate windows is 1/3 of the size at level 1 and the size of the search windows is bigger than that at level 1, which is selected according to the deformation degree. The initial axial and lateral displacement estimates of level 2 are inherited from the output of level 1 and bilinear interpolated to the finer scale. The search windows located at the point according to the output and the center of calculate windows.

**Figure 2:**
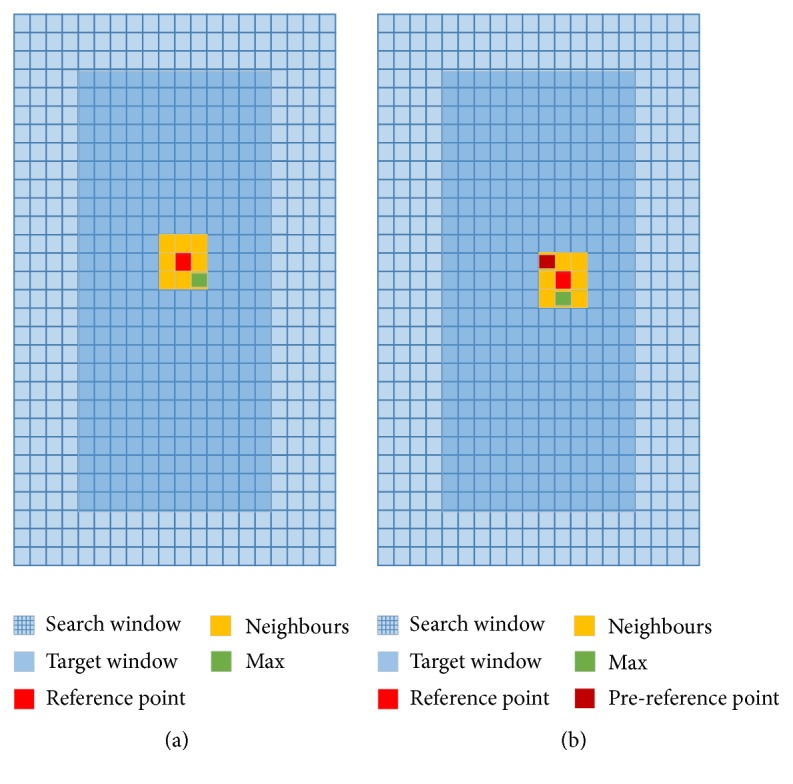
Level 2 search strategy. (a) Set the initial center point of search window to be a reference point and then find the max in its neighbours according to the correlation coefficient. (b) Propagate the reference and its neighbours and then continue to calculate the correlation coefficient.

The so-called “following tracking” strategy (see Figure 2) is used to decide the search direction at each window [22]. First, set the initial center point of search window to be a reference point, and then set the reference point's immediate neighbours to be the search points; after that we need to calculate the correlation coefficient of calculate windows and search windows at each search point. The point which has the highest correlation coefficient will be the next reference point; the search points then propagate to the current reference point's neighbours, and so on. When the reference point occurs at a fixed position or out the range of search window, the calculation in this window is completed, and the axial and lateral displacement estimation is according to the position of the final reference point. Chen et al. used a multiresolution search method in this level; we removed it and increased the numbers of the reference point's neighbours to obtain a more accurate result. We suggested that the increment of computational complexity in this level is meaningful.

However, this time the searches are not independent; there is another delivering strategy that is used to deliver displacement estimations from one window to the next window which on the same column. The next window's initial reference point is no longer the center point of search window, but the position according to the output of the current window.

The advantage of the searching strategy above is that we can find the point which matches the highest correlation coefficient quickly with lower computational complexity. Moreover, the strategy has a good error-correction mechanism to ensure displacement continuity in both axial and lateral direction.

#### 2.2.3. Level 3 Search

The calculate windows and search windows are the smallest in coarse search and this process should finish the whole frame search. The size of calculate windows should be 1/2 of the size at level 2, and level 3 search stops when the calculate windows cover the whole frame. Similar as in level 2, we also need to interpolate the displacement results in level 2 to level 3 with finer scale. And we use the same search strategy to obtain the axial and lateral displacement in level 3 [22]. Since we do not need further refinement of the lateral displacement, and in order to avoid shifting the signals laterally too much, we extract the significant part of the lateral displacement and only shift the RF signal within that region. The lateral displacement should be smoothed and be considered as the final lateral displacement. There are many kinds of the smooth method; we suggest using the Savitzky-Golay filtering method.

### 2.3. Fine Estimation

#### 2.3.1. Displacement Estimation

The initial axial and lateral displacement estimation over the whole RF data can be bilinear interpolated from the coarse estimation from level 3 search. A modified phase zero algorithm is proposed for the fine displacement estimation [13].

Let us denote *x*_1_ and *x*_2_ to be the 1D windowed RF signals before and after compression of the tissue. In elastography, the postdeformation RF data is considered to be a compressed and time-shifted vision of the predeformation signals, and the signal compression can be neglected. Thus, *x*_2_ can be expressed as

(2) $$\begin{array}{c} x_{2}\left(\;t\;\right)=x_{1}\left(\;t+\tau \;\right). \end{array}$$

In general, the correlation between two signals can be calculated from the cross-correlation function as follows:

(3) $$\begin{array}{c} \langle \;a,b\;\rangle \langle \;t\;\rangle =\overset{\infty }{\underset{-\infty }{\int }}a^{*}\left(\;t^{\prime }\;\right)b\left(\;t^{\prime }+t\;\right){dt}^{\prime }. \end{array}$$

Consequently, the signal's autocorrelation function can be a time-shifted modification of the cross-correlation function

(4) x1,x2t=∫−∞∞x1∗t′x2t′+tdt′=∫−∞∞x1∗t′x1t′+t+τdt′,∫−∞∞x1∗t′x1t′+t+τdt′=∫−∞∞x1∗t′x1t′+t+τdt′=x1,x1t+τ,x1,x2t=x1,x1t+τ.

Since the maximum of the autocorrelation equals the maximum of the cross-correlation, the conventional cross-correlation determines this maximum to estimate the time shift. When we return the baseband signals to analytic signals, the phase *φ*(*t*) of the correlation function of the analytic signals *x*_1+_(*t*) and *x*_2+_(*t*) has an identical root

(5) φ−τ=0,φt=arg⁡x1+,x2+t,x1+t=exp⁡jϖ0tx1t,x2+t=exp⁡jϖ0tx2t.

Using the Newton iteration, we can figure out the root of *φ*(*t*) to find the time shift estimation

(6) τ0,0=0,τk,0=τk−1,Nτk,l=τk,l−1−φtnφ′tn≈τk,l−1−φtnϖ0=τk,l−1−arg⁡x1+,x2+τk,l−1ϖ0.

Then replace it by a sum of oversampled signals

(7) τk,l=τk,l−1−1w0arg⁡exp⁡jϖ0τk,l−1·∑τk,l−1−T/2τk,l−1+T/2x1∗tx2t−τk,l−1,

where *τ*_*k*,*l*_ denotes the displacement estimation in the *k*th window after iterating for* l* times and* T* denotes the calculation window size.

In original phase zero-crossing estimation, the displacement estimation of the previous window will be used as the initial value for the next window [13]. The error of one window will propagate to the remaining windows along the RF line. This can cause large accumulated errors in the whole frame. On the other hand, this algorithm is based on the assumption that the lateral displacement in the tissue is negligible. However, tissue will be deformed in both the axial and the lateral directions when freehand compression is applied [21]. Lateral displacement should be considered when estimating the whole displacement field.

We propose a modified algorithm to solve the problems described above. We get the coarse estimation including axial and lateral displacement in the previous coarse calculation, so the iteration between the neighbouring windows can be simplified comparing with 1D elastography method. And the lateral deviation will be applied to the computational process. The calculation is independent of each window, so it makes it easier to take this algorithm to be executed based upon CUDA (Compute Unified Device Architecture)

(8) τ0,0=0,τk,0=τk,coarse′,τk,l=τk,l−1−1w0argexpjϖ0τk,l−1·∑τk,l−1−T/2τk,l−1+T/2x1∗tx2,shiftt−τk,l−1.

The initial value of each window is replaced by the coarse axial displacement *τ*_*k*,coarse_′ on current window. And take the lateral shift on *x*_2_(*t*) to be *x*_2,shift_(*t*).

#### 2.3.2. Strain Estimation

Strain is defined as the gradient of the displacement. Here we only calculate the axial strain and the least-squares strain estimator (LSQSE) is used, which employs a piecewise linear curve fitting [23]. After the axial displacement field is obtained, the strain map may be modeled as

(9) $$\begin{array}{c} u\left(\;i\;\right)=az\left(\;i\;\right)+b, \end{array}$$

where *u* denotes the axial displacement and *z* is the tissue depth. The constants *a* and *b* are the coefficients to be estimated, and *a* represents the local strain. Transform the *z* to matrix form

(10) $$\begin{array}{c} \underline{u}=A\left[\begin{array}{c} a\\ b \end{array}\right]=\left[\begin{array}{cc} z\left(1\right) & 1\\ z\left(2\right) & 1\\ \cdots & 1\\ \cdots & 1\\ z\left(N\right) & 1 \end{array}\right]∙\left[\begin{array}{c} a\\ b \end{array}\right]. \end{array}$$

The least square solution is given by

(11) $$\begin{array}{c} \left[\begin{array}{c} \hat{a}\\ \hat{b} \end{array}\right]=A^{T}{\left[\;AA^{T}\;\right]}^{-1}\hat{u}, \end{array}$$

where $\hat{a}$ and $\hat{b}$ denote the estimation of *a* and *b*, respectively.

### 2.4. Simulation

**Figure 3:**
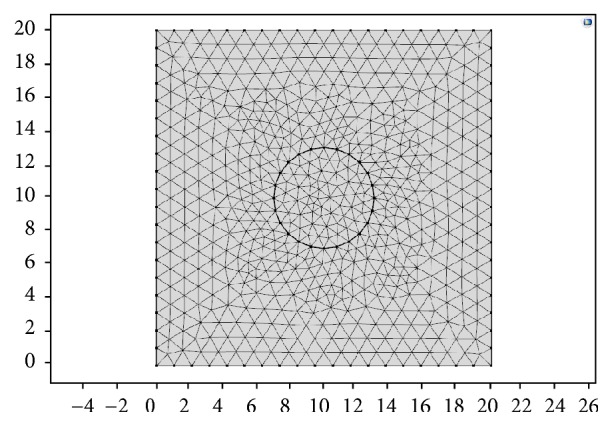
Heterogeneous finite element model simulates the stiffer circular inclusion.

A FEM (finite element model) phantom is simulated using Commercial FEM software COMSOL Multiphysics 5.0 (COMSOL USA). FIELD II software is used for ultrasound simulations. The size of rectangle background of the 2D model is 20 mm × 20 mm, with a stiffer circular inclusion in the center. The radius of the inclusion is 3 mm. Triangular mesh was generated and refined automatically by COMSOL (see Figure 3). Smooth displacement and strain field can be obtained for further RF signal simulation. The inclusion and its background have same density of 1000 kg/m3 and Poisson's ratio of 0.495. Young's modulus of the inclusion is 100 kPa, which is multiple times stiffer than the background. In this study, we set strain contrast between lesion and background to be 5, so that there will be a clear boundary and CNRe can be easily used for performance comparison. The phantom is compressed by uniaxial compression with axial displacement set to 0.3 mm–0.8 mm (i.e., the axial strain is 1.5%–4.0%). The bottom of the phantom is fixed.

A 192-element linear array transducer (64 active elements) with a center frequency of 7.5 Mhz is simulated. The transducer has a pitch of 0.255 mm and an element height of 5 mm. The element width is equal to the wavelength. The number of scan-lines is 128 and the distance between adjacent lines is equal to the pitch. The transmitting focus is at 30 mm and dynamic focusing with focal zones step by 1 mm is used for receiving focus. The image zone has a width of 20 mm and a depth of 20 mm. The speed of sound is assumed to be 1540 m/s and the sampling frequency of the RF signals is 120 Mhz. The original scatters are randomly distributed in the image zone with random scattering amplitude. The predeformation RF signals are simulated with FIELD II using the parameters described above [20, 24]. The new scatters positions after compression are calculated according to COMSOL simulation. The postdeformation RF signals are then simulated with FIELD II using the new scatterer distribution.

## 3. Results and Discussion

Ultrasound RF data was generated using computer simulation. With the synthetic RF data, the displacement and strain distribution were calculated using the modified 2D multiresolution hybrid elastography. Comparison was made between the proposed method, the original 1D elastography and the 1D elastography using downsampled (d-s) baseband signals. Since the modified method has a downsampled step, we make a downsampled version of 1D elastography to compare with the proposed method. To achieve that, we use the same processes as the 1D elastography method to get envelop signals, and the envelop signals will be then downsampled and further 1D elastography calculations will be performed on the downsampled envelop signals.

**Figure 4:**
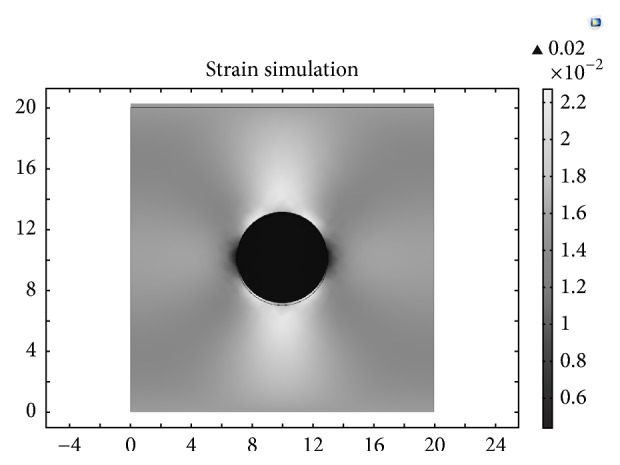
Strain map simulated by COMSOL as the ground truth.

**Figure 5:**
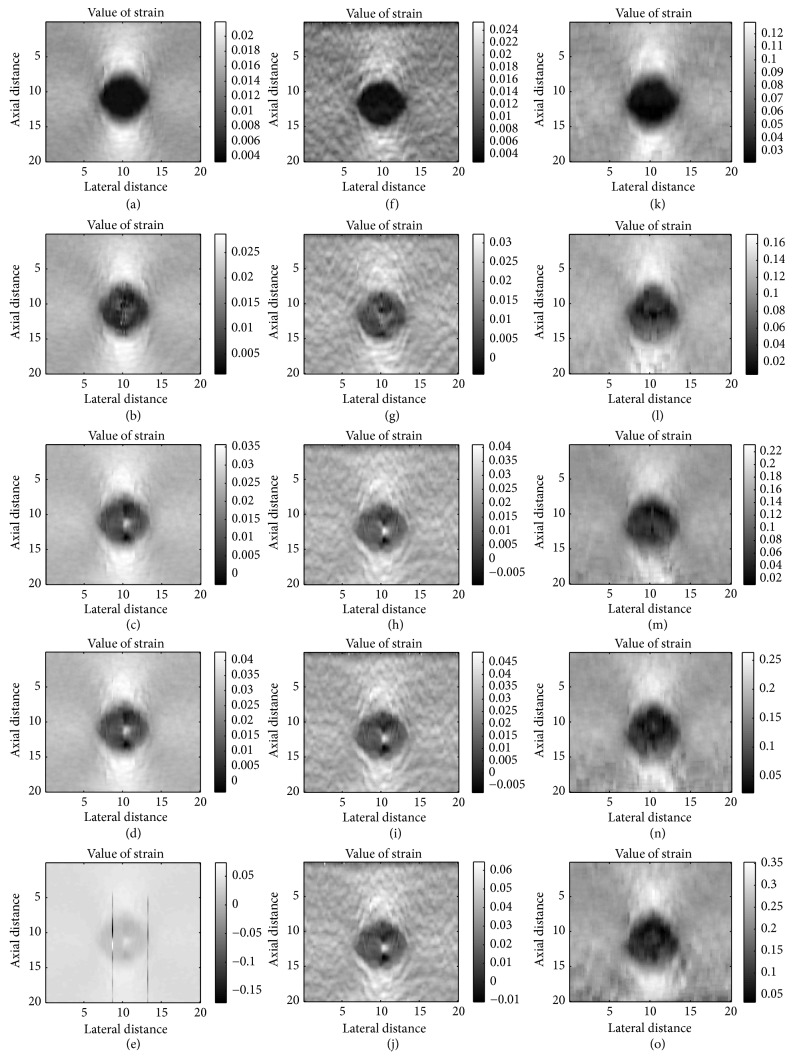
Strain maps of three different algorithms. Different strain levels as 1.5%, 2.0%, 3.0%, 3.5%, and 4.0% are applied at each row. The first column ((a)–(e)), the second column ((f)–(j)), and the third column ((k)–(o)) show, respectively, the strain map obtained by original 1D elastography and original 1D elastography with downsampled and modified 2D multiresolution hybrid elastography.

Figure 4 shows the theoretical strain maps using finite element analysis in COMSOL as the ground truth.  Figure 5 presents the estimated strains obtained by three algorithms for the computational phantom under different applied strain. The direct viewing of the result shows that 1D elastography is slightly better under low strain level; the reason is that the 1D method build on the whole RF data and the iterative calculation is more complete and precise under low strain level. The performance of the algorithm we proposed is similar to 1D algorithm when low strain is applied but much better under higher strain level. As we can see in the first two columns, some error appeared in the center of the result when strain level increased, and there are two error lines in 1D elastography (Figure 5(e)) because of the lacking consideration of lateral displacement and iterative calculation between two windows.

**Figure 6:**
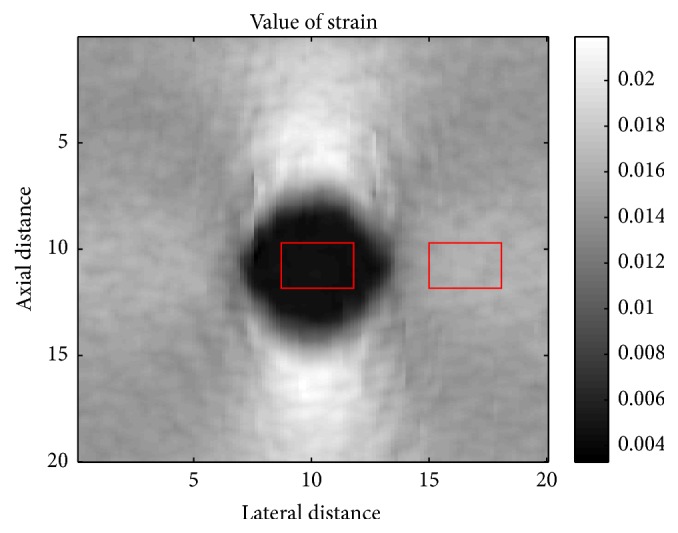
The extra area of inclusion and background for the calculation of CNRe and the average estimated strain rate.

To quantify the performance of different algorithms, CNRe is calculated at each strain level and we use the binary strain image to measure the accuracy of each algorithm [25]. The definition of the CNRe is provided in the following:

(12) $$\begin{array}{c} {CNR}_{e}=\frac{2{\left(s_{1}-s_{2}\right)}^{2}}{\sigma_{s_{1}}^{2}+\sigma_{s_{2}}^{2}}, \end{array}$$

where *s*_1_ and *σ*_*s*_1__^2^ denote the mean and the variance of strain estimation in the inclusion and *s*_2_ and *σ*_*s*_2__^2^ are the mean and the variance of strain estimation in the background (see Figure 6). The larger the CNRe value, the better the clarity of the results.

**Figure 7:**
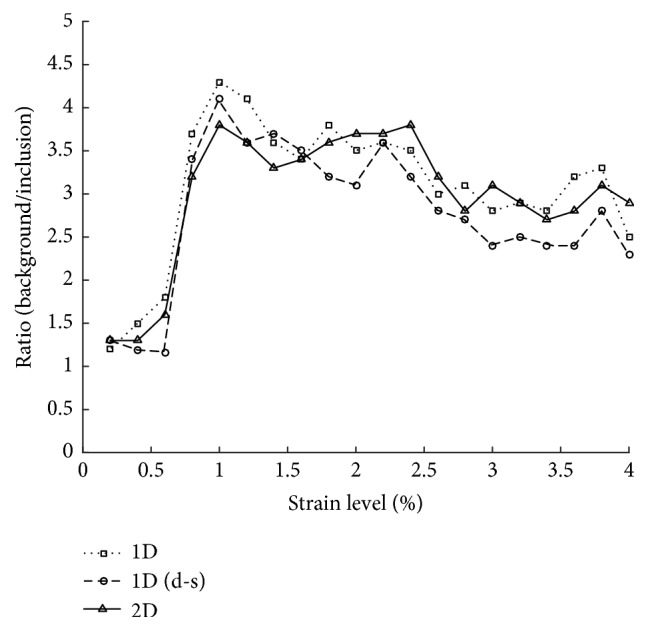
The ratio of average estimated strain rates of the inclusion and the background.

We have made a record of the average estimated strain rate for both inclusion and background for comparison purpose. The ratio of *s*_1_ and *s*_2_ previously mentioned is shown directly in Figure 7. We can see that the three different algorithms have shown similar average strain ratio between lesion and background in different strain level.

**Figure 8:**
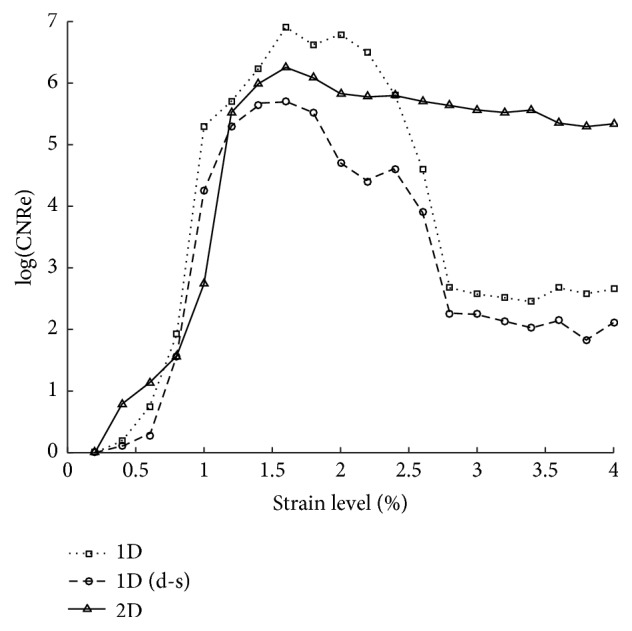
The CNRe results of three different algorithms.

We make a complete test with three algorithms under different strain level range 0.2% to 4%. Same as 1D algorithms, the CNRe of our algorithms is too low to distinguish between the lesion and background when strain level is lower than 0.8%, which means the noise on strain estimation will blur the boundary between lesion and background. And the CNRe of the modified 2D multiresolution hybrid elastography algorithm is much higher than the other two algorithms at high strain level (see Figure 8). We can see that the original 1D elastography has a slight better result in low strain level; the reason is that the 1D method build on the whole RF data and the iterative calculation is more complete and precise under low strain level. The performance of the algorithm we proposed is similar to 1D algorithm when low strain is applied but better under higher strain level.

**Figure 9:**
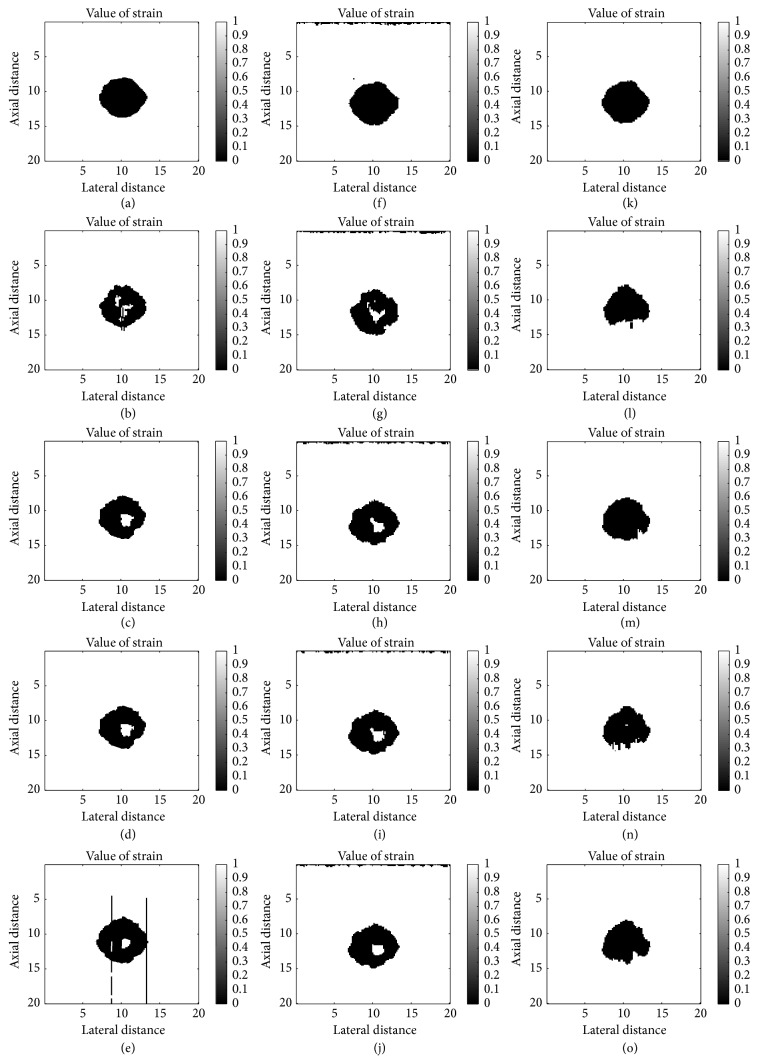
Strain binary images of three different algorithms. Different strain levels as 1.5%, 2.0%, 3.0%, 3.5%, and 4.0% are applied at each column. The first column ((a)–(e)), the second column ((f)–(j)), and the third column ((k)–(o)) show, respectively, the strain binary map obtained by the corresponding strain map using 1D elastography, downsampled 1D elastography, and the proposed 2D elastography method.

**Figure 10:**
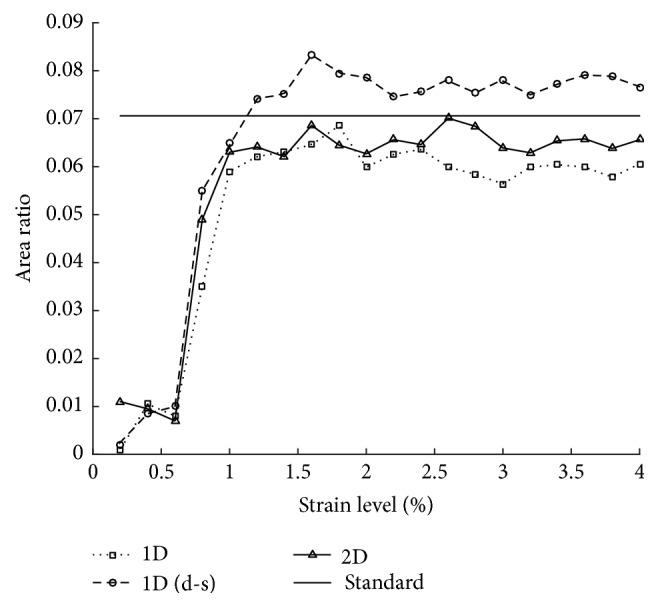
The inclusion area ratios results of three different algorithms, where the standard denotes the inclusion area ratio in FEA model.

The strain binary images (see Figure 9) are obtained by thresholding the strain map. We set the threshold at the same level and plot the binary images, respectively. It is an immediate way to see the accuracy of each strain image. We set the average estimated strain rate for whole phantom as the threshold of the binary images. Additionally, the inclusion area ratios are calculated and compared with the ground truth of FEA result. Then we calculate the inclusion area ratio as the numbers of black pixels and all pixels in the image. The standard denotes the inclusion area ratio in FEA model and results of calculated strain ratio from three methods are shown in Figure 10. The inclusion area ratio of the new method is closer to the standard than other methods.

In the algorithm we proposed, the lateral resolution depends on the amount of the windows in level 3 search. The lateral resolution increased with the increasing of the amount until the resolution is equal to the sampling frequency of the RF data. The axial resolution is determined by the physical size of transducer such as the kerf of the array element. The purpose of the consideration of lateral displacement in our algorithm is not to improve the later resolution but to compensate for the interference of lateral offset and get higher accuracy in axial strain image.

All the algorithms were executed with an Intel(R) Core(TM) i7-4790K CPU @2.40 GHz 8.00 GB RAM, and MATLAB 2014b was used for implementing and testing them on Windows operation system. The execution times of the different methods are shown in Table 1. The modified 2D multiresolution hybrid elastography algorithm has a shorter time than the other algorithms.

## 4. Conclusion

A modified 2D multiresolution hybrid method has been proposed in this paper. Using finite element model phantom under different strain levels, we have shown that the new algorithm can achieve better CNRe comparing with different methods including original 1D elastography method and original 1D elastography method with downsampled (d-s). These results demonstrate that the method we suggested is robust and accurate when high level strain is applied. The result of execution time has shown that the new framework has a higher efficiency that it may well be more suitable for real-time application in clinical practice. The limitation of our algorithm is that, to a great extent, the accuracy of the final result is determined by the output of coarse estimation. The strain image calculated by our algorithm is slightly worse than original 1D elastography under low strain level. In this study, we use simulated data to compare the performance of the proposed method with other methods in different strain level, since ground truth of strain map can be easily obtained. In our next study, we will test our method using phantom and in vivo data.

## Figures and Tables

**Table 1:** Execution times of three different algorithms.

Method	1D	1D with downsampled	Modified 2D
Times (s)	10.2	2.3	1.8
